# Adeno-Associated Vector-Delivered CRISPR/*Sa*Cas9 System Reduces Feline Leukemia Virus Production In Vitro

**DOI:** 10.3390/v13081636

**Published:** 2021-08-18

**Authors:** A. Katrin Helfer-Hungerbuehler, Jimit Shah, Theres Meili, Eva Boenzli, Pengfei Li, Regina Hofmann-Lehmann

**Affiliations:** Clinical Laboratory, Center for Clinical Studies, Department of Clinical Diagnostics and Services, Vetsuisse Faculty, University of Zurich, 8057 Zurich, Switzerland; jimitshah123@gmail.com (J.S.); tmeili@vetclinics.uzh.ch (T.M.); eboenzli@vetclinics.uzh.ch (E.B.); pengfei.li@uzh.ch (P.L.); rhofmann@vetclinics.uzh.ch (R.H.-L.)

**Keywords:** feline leukemia virus, FeLV, CRISPR, *Sa*Cas9, AAV, provirus, gene editing, cat

## Abstract

Feline leukemia virus (FeLV) is a retrovirus of cats worldwide. High viral loads are associated with progressive infection and the death of the host, due to FeLV-associated disease. In contrast, low viral loads, an effective immune response, and a better clinical outcome can be observed in cats with regressive infection. We hypothesize that by lowering viral loads in progressively infected cats, using CRISPR/*Sa*Cas9-assisted gene therapy, the cat’s immune system may be permitted to direct the infection towards a regressive outcome. In a step towards this goal, the present study evaluates different adeno-associated vectors (AAVs) for their competence in delivering a gene editing system into feline cells, followed by investigations of the CRISPR/*Sa*Cas9 targeting efficiency for different sites within the FeLV provirus. Nine natural AAV serotypes, two AAV hybrid strains, and Anc80L65, an in silico predicted AAV ancestor, were tested for their potential to infect different feline cell lines and feline primary cells. AAV-DJ revealed superior infection efficiency and was thus employed in subsequent transduction experiments. The introduction of double-strand breaks, using the CRISPR/*Sa*Cas9 system targeting 12 selected FeLV provirus sites, was confirmed by T7 endonuclease 1 (T7E1), as well as Tracking of Indels by Decomposition (TIDE) analysis. The highest percentage (up to 80%) of nonhomologous end-joining (NHEJ) was found in the highly conserved *gag* and *pol* regions. Subsequent transduction experiments, using AAV-DJ, confirmed indel formation and showed a significant reduction in FeLV p27 antigen for some targets. The targeting of the FeLV provirus was efficient when using the CRISPR/*Sa*Cas9 approach in vitro. Whether the observed extent of provirus targeting will be sufficient to provide progressively FeLV-infected cats with the means to overcome the infection needs to be further investigated in vivo.

## 1. Introduction

The impact of retroviral infections on human and animal health is of major scientific and public interest. Although the number of HIV-related deaths has decreased continuously since its peak in 2005, curative treatments for retroviral infections are nonexistent, and it is estimated that between 480,000 and 1 million people died from AIDS-related illnesses worldwide in 2020 [1]. Understanding the underlying biology of retroviral infections is fundamental toward developing successful antiviral strategies, such as preventive and therapeutic measures. The persistence of retroviral reservoirs in the cellular genome is challenging and has not been directly targeted by commonly used antiretroviral therapies (ARTs) [2] against HIV. Thus, animal models are needed to investigate host–virus interactions and to evaluate new therapies.

**Figure 1:**
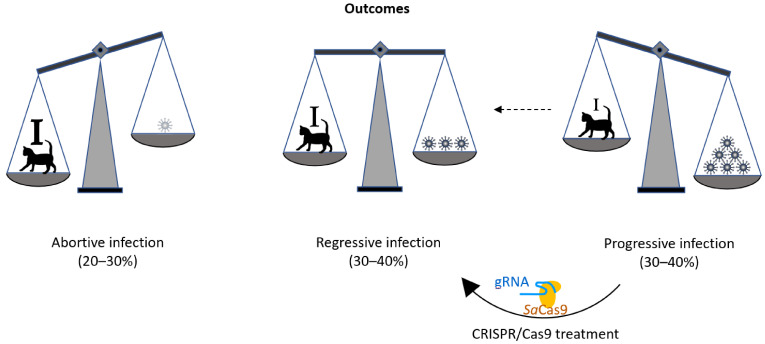
Schematic drawing of the outcomes of FeLV. In the course of FeLV infection, the virus/provirus load often corresponds to the disease outcome. Whereas during an abortive infection the cat’s immune response successfully eliminates viral proliferation (strong immune system: I), during progressive infection viral loads are constantly high (lack of effective FeLV-specific humoral and cellular immunity: i). During regressive infection, an effective immune response (marked with **I**) can control FeLV, often during the entire lifetime of the cat, thereby omitting FeLV-related disease and death. It is the goal of this study to develop the tools to shift the outcome of FeLV infection from progressive to regressive, with the help of a CRISPR/*Sa*Cas9 treatment that reduces the FeLV proviral loads in the cats’ cells and helps the immune system to overcome viremia.

As in humans, retroviral infections are prevalent in diverse animal species, including the domestic cat. The prevalence of feline leukemia virus (FeLV; a gammaretrovirus) infection varies greatly, depending on host sex, age, health, environment, and lifestyle [3]. FeLV in cats is a well-established animal model for tumor and retroviral research that has been used for nearly 50 years [4]. After FeLV exposure, most cats develop a regressive FeLV infection, characterized by an early and efficient immune response, mediated by neutralizing antibodies [5,6] and FeLV-specific cytotoxic T lymphocytes (Figure 1, [7,8,9]). This remarkable feature of FeLV infection in cats yields a great opportunity to study effective antiretroviral immune responses. However, approximately one-third of cats exposed to FeLV develop progressive FeLV infections with persistent viremia; these animals lack effective FeLV-specific humoral and cellular immunity [10,11]. These progressively infected cats develop fatal FeLV-associated diseases, including lymphoma, leukemia, immune suppression, and anemia, and they die within a few years of infection [12,13]. Results from an earlier study demonstrated that the different infection outcomes are associated with differences in viral RNA and provirus tissue loads [13]. It is very rare, but possible, for cats to raise a protective immune response and overcome persistent viremia on their own, even months after infection. Such a full recovery from viremia has been observed in one cat, more than one year after the onset of infection [14]. This proves that under the right circumstances, recovery from an established progressive retroviral infection is possible. We hypothesize that the reduction of the proviral load in cats with progressive infection can tip the virus–host balance, in favor of the immune system of the cat; an effective immune response may be raised and help the cat to overcome persistent viremia and evade subsequent infection-related disease (Figure 1).

To date, no treatment regimen is known to reliably cure either progressive FeLV infection in cats or other retroviral infections. Thus, new classes of therapeutics are being developed, including the investigation and application of gene therapy. Adeno-associated virus (AAV)-based vectors represent promising candidates for therapeutic gene transfer, due to their lack of involvement in human diseases and their display of low immunogenicity [15]. AAV has been used as a vector for over 20 years [16] in more than 100 clinical trials. With increasing clinical elaboration, some limitations of in vivo gene transfer, using AAV-vectors, have been recognized. Previous infections with natural AAVs, which may have similar or even identical capsids, compared to the gene therapy vectors, can result in the production of cross-reactive or specific anti-AAV neutralizing antibodies (NAb), which partially, or even completely, block transduction of the target tissue [17,18,19]. Thus, the previous survey of pre-existing NAb to AAV in domestic cats laid a foundation for future studies, leading towards gene therapy in this outbred species [20]. Compared with the prevalence of NAb against AAV in humans, the NAb in cats were found less frequently, ranging from 5–28%. Even lower AAV-binding Ab with neutralizing activity were detected in a study in cats living in the US [21]. A low prevalence of NAb against AAV in cats supports the usability of AAV for gene therapy in this species. However, potentially preexisting NAb to AAV will need to be considered for individual in vivo gene therapies in cats.

The fact that FeLV was detectable in the form of viral RNA and DNA in all 27 examined tissues of viremic cats has important implications for the future use of AAVs as gene therapy vectors in FeLV-infected felids [13]. Although a huge variety of AAV serotypes have been described in the literature, so far, few studies have been performed in cats, focusing primarily on AAV1, AAV8, AAV9, AAVrh8, AAV-B1, and AAV-AS [22,23,24,25,26,27,28,29]. Unfortunately, none of these, so far studied, AAV serotypes fit the broad cell tropism of FeLV. Thus, one goal of the present study was to investigate additional AAV vectors for their efficiency to infect different feline cell lines.

Apart from finding a suitable gene therapy vector to be used in cats, a method was developed to lower FeLV proviral loads. In the search for a tool to aid cats in overcoming progressive FeLV infection, by lowering proviral and viral RNA loads, a recently developed gene-editing technology was used. We employed the RNA-guided Cas9 nuclease [30,31,32] from the type II prokaryotic clustered regularly interspaced short palindromic repeats (CRISPR) adaptive immune system [33,34,35]. Its efficiency, accuracy, and simplicity make it an extremely powerful tool which, when applied to the FeLV model, provides an exceptional opportunity to investigate new antiretroviral strategies in vitro, ex vivo, and in vivo. The RNA-guided endonuclease Cas9 from *Staphylococcus aureus* (*Sa*Cas9) was selected for the detection, cleavage, and subsequent inactivation of the FeLV provirus, due to its small size (1053 amino acid [aa] versus 1368 aa) and the genome editing efficiencies, similar to *Streptococcus pyogenes* Cas9 (*Sp*Cas9) [36].

The CRISPR/Cas9 system has a conceptually straightforward design: a single guide RNA (sgRNA) directs the Cas9-mediated endonuclease activity to the site of interest. The only constraint is the requirement of a specific protospacer adjacent motif (PAM) on the target template immediately following the sgRNA target sequence. Different Cas9 orthologues have specific PAM sequence requirements. Upon complementary base pairing of the sgRNA to the DNA target, which is PAM dependent, Cas9 produces a targeted double-strand break in the DNA [37]. The efficacy of directing Cas9-mediated, DNA-modification varies between sgRNAs. Reasons for this are manifold and include suboptimal sgRNA design [38], chromatin structure, and genomic elements [39,40]. Subsequently, this double-strand break can either be repaired by the endogenous cellular repair machinery by nonhomologous end-joining (NHEJ), leading to local insertions and/or deletions (indels), or via homology-directed repair when a donor template is available.

Overall, the goals of the present study were to develop the necessary tools to reduce the FeLV proviral load in cats and, thus, give the development of an effective immune response a chance. For this, we characterized the infectivity of various AAV serotypes in vitro, in order to identify AAVs with broad cell tropism, corresponding to that of FeLV. In the second step, 12 FeLV sgRNAs were identified in silico, three in each region of FeLV, and characterized and compared in their efficiency to target and reduce FeLV provirus reservoirs in vitro.

The use of CRISPR/*Sa*Cas9 for the deletion or deactivation of FeLV proviruses in the cellular genome offers the possibility to reduce retroviral reservoirs in the genome and, with this, the potential for a cat to switch from a progressive to a regressive infection outcome (Figure 1).

## 2. Materials and Methods

### 2.1. Cell Culture

The following cell lines were used in this study: Crandell–Rees feline kidney cells (CRFK, ATCC CCL-94), feline embryonic fibroblasts (FEA) [41], feline astrocytes (PG-4, ECACC 94102703), cat lung epithelial cells (AK-D, ECACC 89071903), feline mammary carcinoma cells (MT, ECACC 12122001), feline tongue fibroblast-like cells (Fc3Tg, ECACC 90073002), feline macrophage cells (Fcwf-4, ATCC CRL-2787), human embryonic kidney cells (HEK-293, ATCC CRL-1573), and Mus dunni tail fibroblasts (MDTF, ATCC CRL-2017, non-infectable with FeLV). Details on the growth medium can be found in the Supplementary Table S1.

Peripheral blood mononuclear cells (PBMCs) were isolated from left-over material of heparin anticoagulated blood samples, submitted to our laboratory for routine diagnostic purposes, unrelated to this study; no additional samples or volumes were collected for the present study. PBMCs were Ficoll-Hypaque purified (Histopaque^®^-1077, Sigma-Aldrich, Buchs, Switzerland), as described [42]. Isolated PBMCs were grown at a density of 1 × 10^6^ cells/mL in a 96 well plate (details on the media in the Supplementary Table S1). At the initiation of the culture, expression of the IL-2 receptor was induced with 1% phytohemagglutinin (Gibco, Thermo Fisher Scientific, Waltham, USA). One day later, recombinant human interleukin-2 (IL-2; Sandoz Pharmaceuticals AG, Cham, Switzerland) was added to a final concentration of 1 ng/mL. On day 2, PBMCs were infected with AAV vectors. All cell lines were cultured in an incubator at 37 °C infused with 5% CO_2_.

### 2.2. AAV Vectors and Their Infectivity In Vitro

**Figure A1:**
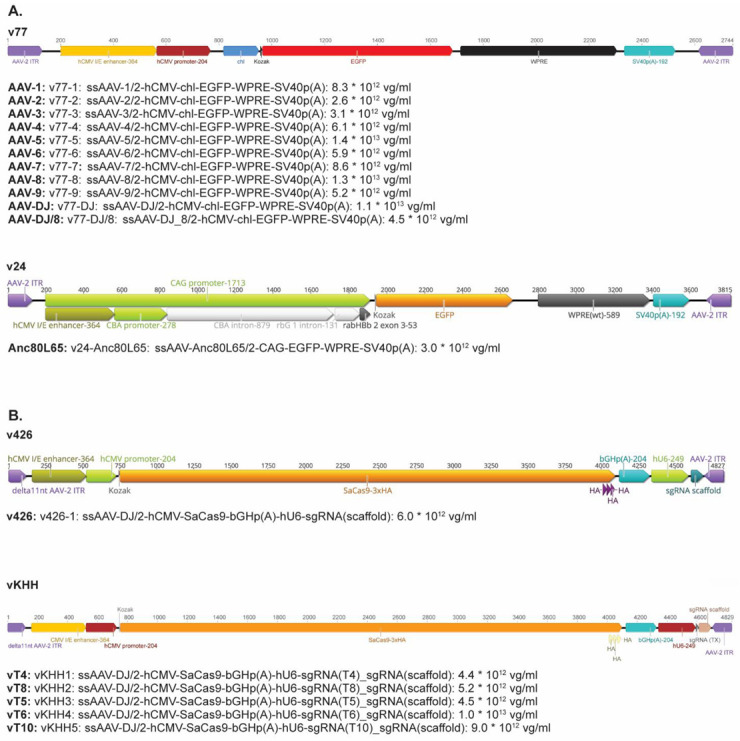
Schematic representation and concentration of the AAV vectors used in this study. (**A**). AAV vectors used for the determination of the tropism. (**B**). Gene therapy vectors.

Nine natural AAV serotypes (AAV1-9), 2 AAV hybrid strains (AAV-DJ and AAV-DJ/8), and the ancestral Anc80L65 vector were used [43]. All AAV vectors contained the reporter gene enhanced green fluorescent protein (EGFP), under the transcriptional control of the human cytomegalovirus (hCMV) immediate/early gene promoter/enhancer fragment hCMV (Appendix A, Figure A1A). The only exception was the Anc80L65 vector: in this vector, the EGFP is under the control of the CAG promoter. All AAV vectors were constructed, purified, and quantified by the Viral Vector Facility (VVF) at the University of Zurich, Switzerland. AAV vectors were stored at −80 °C until utilized.

In order to investigate the infectivity of different AAV serotypes in cell lines (CRFK, FEA, PG-4, AK-D, MT, Fc3Tg, Fcwf-4, FetJ, and HEK-293), as well as feline PBMCs, a series of AAV infectivity ratios to cells (multiplicity of infection (MOI): 100 to 100,000 viral genomes (vg)/cell), were tested (always in duplicates (*N* = 2)). HEK-293 served as control cell line, which had previously been shown to be infectable. Depending on the cell line, 5 × 10^4^–1 × 10^5^ cells/mL, as enumerated manually using a Neubauer counting chamber (Semadeni, Ostermundigen, Switzerland), and 1 × 10^6^ cells/mL PBMCS, counted using a Sysmex XT-2000iV (Sysmex Corporation, Kobe, Japan), were cultured overnight in a 96-well tissue culture. Infection was followed by fluorescence microscopy between days 2 and 6. Fluorescent images were captured using an inverted fluorescent microscope (EVOS FL, Life Technologies, Waltham, MA, USA). Quantification of the mean fluorescence intensity of the raw microscopy pictures was performed using ImageJ 1.53 k, an open source image processing and analysis tool [44]. The signal strength ranges between 0 (black) and 255 (white).

### 2.3. Infection of Cells with FeLV-A/Glasgow-1 and FeLV Antigen Quantification

Various cell lines were infected with undiluted FeLV-A/Glasgow-1 supernatant from FeLV-A/Glasgow-1-infected FEA cells. FEA, CRFK, PG-4, AK-D, MT, and M. dunni cells (negative control) were seeded at a density of 200,000 cells per well on a 6-well plate. Two wells contained uninfected cells as negative controls. After 24 h, the cells reached a confluency of 50–60%. Infection time was two hours, followed by a washing step with the original growth medium (Supplementary Table S1). The medium was changed 24 h after infection, and the cells were passaged in T25 flasks after 48 h. After 72 h, cells were transferred to T75 flasks. On day 3 and day 7, the presence of FeLV p27 antigen was determined in the supernatant, by sandwich enzyme-linked immunosorbent assay (ELISA), as described [45]. ELISA results were calculated as a percentage, after normalization to the positive control (culture supernatant of FL-74 feline lymphoblastoid cell line permanently infected with FeLV), which was assayed on every plate. Values above 4% of this positive control were considered positive [46].

### 2.4. Gene Editing Technology and Target Selection

**Figure 2:**
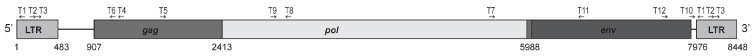
Locations of the 12 FeLV sgRNAs (T1 to T12) in the FeLV-A/Glasgow-1 genome (GenBank: KP728112.1). The three genes, *gag* (group-specific antigen), *pol* (polymerase), and *env* (envelope) are flanked by the LTRs (long terminal repeats). Three target DNA sequences were chosen in *gag* (T4–T6), *pol* (T7–T9), *env* (T10–T12), and LTR (T1–T3), each marked with an arrow. The size of the genes and the LTRs is proportional to the genome size; arrows mark the location of the target, but their size is not to scale. Numbers below the schematic of the genome represent the beginning and end of the LTRs, as well as the beginning of each gene.

The recently developed gene editing technology CRISPR/*Sa*Cas9 was employed to investigate its potential in cutting selected target sites within the FeLV provirus. Due to its smaller size and genome editing efficiencies, similar to *Sp*Cas9, *Sa*Cas9 was chosen for gene editing of FeLV. Twelve target DNA sequences, T1 to T12, were selected within the FeLV provirus, based on in silico algorithm prediction, using the software Geneious and sgRNA Scorer 2.0, following the rules for U6 PolIII transcription and the *Sa*Cas9 PAM recognition domain (NNGRRT, Figure 2). *Sa*Cas9 enzyme cleavage stereotypically occurs 3 bp upstream of the PAM site [36]. Minimalizing the off-target ratio and increasing target specificity were the main goals when choosing target sites equally distributed in all three genes of FeLV (T4–T6 in *gag* (group-specific antigen), T7–T9 in *pol* (polymerase), and T10–T12 in *env* (envelope)), and T1–T3 in the long terminal repeat (LTR, Figure 2, Table 1).

All 12 sgRNAs were cloned into the plasmid px601-GFP, a gift from Yuet Wai Kan (Addgene plasmid # 84040; http://n2t.net/addgene:84040 accessed on 21 June 2017; RRID:Addgene_84040, [47]). In brief, *Sa*Cas9-sgRNA plasmids were constructed using *Sa*Cas9 guides, flanked by Bsa1 sites ligated into Bsa1 cut px601-GFP. All the designed plasmids were verified by Sanger sequencing in a commercial laboratory (Microsynth, Balgach, Switzerland).

### 2.5. Transfection

FeLV-A/Glasgow-1-infected cells (200,000 cells; CRFK, PG-4 or FEA) were seeded in duplicates in 6-well plates. The next day, the cells were transfected, when confluency was around 50%, with 2.5 μg plasmid DNA, using Lipofectamine^TM^ 3000 reagent (Invitrogen, Thermo Fisher Scientific), according to the manufacturer’s protocol.

Successful transfection of the *Sa*Cas9-EGFP-TX was confirmed by the microscopic detection of the EGFP expression after two days. Using this protocol, the transfection efficiency obtained was around 5% for CRFK cells and 20–30% for PG4 and FEA cells. Despite optimization (variations in plasmid und Lipofectamine concentrations, electroporation, instead of chemical transfection), the transfection efficiency could not be further improved; therefore, the transfected cells were trypsinized, washed, and collected in PBS before sorting, based on their EGFP-signal, using a FACS analyzer (BD Aria III at the Cytometry Facility, University of Zurich, Switzerland). Duplicates had to be combined to collect >200,000 cells per sample.

### 2.6. Nucleic Acid Extractions and TaqMan Fluorogenic Real-Time PCR Assay

Genomic DNA (gDNA) was extracted from cells using a DNeasy Blood & Tissue Kit (Qiagen, Hombrechtikon, Switzerland), following the manufacturer’s recommendations, with an elution volume of 100 μL per sample. For all nucleic acid extractions, negative controls, consisting of 100 μL of phosphate-buffered saline (PBS), were prepared with each batch to monitor cross contamination.

The gDNA was amplified and quantified on an ABI Prism 7700 sequence detection system (Applied Biosystems), and copy numbers were determined using corresponding standards, generated from serial dilutions of a synthetic oligonucleotide. Negative extraction controls, as well as water controls, served as negative controls. FeLV provirus loads were quantified by TaqMan real-time PCR (U3 region) [48]. As two LTR U3 target sequences are present for each FeLV provirus copy, the number of provirus copies was calculated by dividing the number of copies of U3 (as determined by real-time PCR (qPCR)) by two. This number of provirus copies was then divided by the number of feline albumin (fALB) copies, measured by qPCR, as described previously [49], to determine the provirus copy numbers per cell.

### 2.7. CRISPR Efficiency Analysis by T7 Endonuclease 1 (T7E1) Mismatch Detection Assay and Tracking of Indels by Decomposition (TIDE)

Heteroduplexes representing nucleotide mismatches, as a result of NHEJ after *Sa*Cas9 cleavage, were detected and located by the application of T7E1 [50]. T7E1 is a structure-selective enzyme that detects structural deformities in heteroduplexed DNA (mismatches) and, thus, can be used as a method to evaluate CRISPR-Cas9-mediated gene editing [50]. In order to carry this out, a conventional PCR was performed on gDNA extracted from CRISPR/*Sa*Cas9-treated cells and control cells, with primers spanning each CRISPR/*Sa*Cas9 sgRNA target region (Figure 2). Genomic DNA (gDNA) was extracted from cells using a DNeasy Blood & Tissue Kit (Qiagen), following the manufacturer’s recommendations, with an elution volume of 100 μL per sample. For all nucleic acid extractions, negative controls consisting of 100 μL of phosphate-buffered saline (PBS) were prepared with each batch to monitor cross contamination. Sequences for the forward and reverse primers, as well as the cycling conditions, are depicted in Table 1. Conventional PCR was performed in a thermocycler (PCR-Gradient-Thermocycler, Biometra, Jena, Germany) using Phusion Hot Start II High-Fidelity DNA-Polymerase (Thermo Fisher). The resulting PCR products were visualized on an agarose gel, which was followed by the determination of CRISPR/*Sa*Cas9 cleavage efficiency by T7 Endonuclease 1 (T7E1, BioConcept, Allschwil, Switzerland) digestion, according to the manufacturer’s recommendations. In brief, 200 ng of purified PCR product (MinElute PCR Purification Kit (Qiagen)) of the sgRNA target region was denatured at 95 °C for 5 min and re-annealed by subsequent slow cooling at −2 °C per second to 85 °C, followed by a −0.1 °C per second to 25 °C. In the case of CRISPR/*Sa*Cas9 cleavage, followed by aberrant NHEJ, heteroduplexes were formed (e.g., between mutant and wild-type (WT) amplicons). A T7E1 digestion followed immediately, detecting these structural deformities. The efficiency of T7E1 cleavage was determined by comparing control and experimental samples on 2% agarose gels (Fusion software) and on a fragment analyzer (FA; 5200, Agilent, Santa Clara, CA, USA) using the dsDNA 935 Reagent Kit (Agilent), based on the size of DNA fragments, ranging from 1 to 1500 bp, using the ProSize 3.0 software. Both detection systems compared band intensities between the control and the cut sample.

*Sa*Cas9-mediated NHEJ efficiency was calculated based on the fraction of cleaved DNA:

(1) $$\%\;\mathrm{NHEJ}=100\times (1-\sqrt{1-\frac{\left(a+b\right)}{\left(a+b+c\right)}})$$

with *a* and *b* being the cleaved DNA and *c* being the uncut DNA [51].

The spectrum and frequency of small indels generated by targeted genome editing tools, such as CRISPR/Cas9, can also be determined by two standard capillary sequencing reactions (wild type and edited sample), followed by sequence comparison using an online R based program [52]. Sanger traces were generated by Microsynth, with target-specific PCR products (Table 1), and analyzed with the TIDE webtool ([52]; http://shinyapps.datacurators.nl/tide/ accessed on 16 June 2020). Default parameters were used for the analysis, with the exception of the indel size range, which was set from 10 to 20 bp.

### 2.8. Production, Purification and Quantification of Single-Stranded (ss) AAV Vectors

The sgRNA4, sgRNA5, sgRNA6, sgRNA8, and sgRNA10 were cloned into the plasmid pX601-AAV-CMV::NLS-SaCas9-NLS-3xHA-bGHpA;U6::BsaI-sgRNA (a gift from Feng Zhang (Addgene plasmid #61591; http://n2t.net/addgene:61591 (accessed on 21 June 2017); RRID:Addgene_61591; [36]). These plasmids contain a human codon-optimized *Sa*Cas9, driven by a cytomegalovirus (CMV) promoter, and a U6-driven sgRNA. In brief, plasmids were constructed, using guides flanked by Bsa1 sites ligated into Bsa1 cut #61591. These #61591/*Sa*Cas9 plasmids were propagated, amplified, and purified using a commercial kit, following the manufacturer’s protocols (NucleoBond Xtra Midi, Macherey Nagel, Fisher Scientific AG, Oensingen, Switzerland). All the designed plasmids were verified by Sanger sequencing (Microsynth).

Single-stranded (ss) AAV vectors were produced and purified, as previously described [53,54]. Briefly, HEK 293 cells [55], expressing the simian virus (SV) large T antigen [56] (293T), were transfected by polyethylenimine (PEI)-mediated cotransfection of AAV vector plasmids (providing the to-be packaged AAV vector genome), the AAV helper plasmid p61 (Cell Biolabs, Inc., San Diego, CA, USA; VPK-420-DJ; p61 providing the AAV serotype 2 rep proteins and the cap proteins of AAV-DJ), and adenovirus (AV) helper plasmids pBS-E2A-VA-E4 [57] (providing the AV helper functions) in a 1:1:1 molar ratio.

At 120 to 168 h post-transfection, HEK 293T cells were collected and separated from their supernatant by low-speed centrifugation. AAV vectors, released into the supernatant, were PEG precipitated over night at 4 °C by adding a solution of polyethylene glycol 8000 (8% *v*/*v* in 0.5 M NaCl) and completed by low-speed centrifugation. Cleared supernatant was discarded, and the pelleted AAV vectors were resuspended in AAV resuspension buffer (150 mM NaCl, 50 mM Tris HCl, pH 8.5). HEK 293T cells were resuspended in AAV resuspension buffer and lysed by Bertin’s Precellys Evolution homogenizer, in combination with 7 mL soft tissue homogenizing CK14 tubes (Bertin). The crude cell lysate was DENARASE (c-LEcta GmbH) treated (150 U/mL, 90 to 120 min at 37 °C) and cleared by centrifugation (10 min at 17,000× *g*/4 °C). The PEG precipitated (1 h at 3500× *g*/4 °C) AAV vectors were combined with the cleared cell lysate and subjected to discontinuous density iodixanol (OptiPrep™, Axis-Shield, Kampenhout, Belgium) gradient (isopycnic) ultracentrifugation (2 h 15 min at 365,929× *g*/15 °C). Subsequently, the iodixanol was removed from the AAV vector containing fraction by 3 rounds of diafiltration using Vivaspin 20 ultrafiltration devices (100,000 MWCO, PES membrane, Sartorius, Göttingen, Germany) and 1× PBS supplemented with 1 mM MgCl_2_ and 2.5 mM KCl, according to the manufacturer’s instructions. The AAV vectors were stored aliquoted at −80 °C.

Encapsidated viral vector genomes (vg) were quantified using the Qubit™ 3.0 fluorometer, in combination with the Qubit™ dsDNA HS Assay Kit (both Life Technologies). Briefly, 5 µL of undiluted (or 1:10 diluted) AAV vectors was prepared in duplicate. Untreated and heat-denatured (5 min at 95 °C) samples were quantified, according to the manufacturer’s instructions. Intraviral (encapsidated) vector genome concentrations (vg/mL) were calculated by subtracting the extraviral (non-encapsidated; untreated sample) from the total intra- and extraviral (encapsidated and non-encapsidated; heat-denatured sample). Constructed AAV vectors (vT4 with sgRNA4 [vKHH1], vT5 with sgRNA5 [vKHH3], vT6 with sgRNA6 [vKHH4], vT8 with sgRNA8 [vKHH2], and vT10 with sgRNA10 [vKHH5]) had vector genome concentrations between 4.4 × 10^12^ vg/mL and 1.0 × 10^13^ vg/mL (Appendix A, Figure A1B).

The identity of encapsidated genomes was verified and confirmed by Sanger DNA sequencing of amplicons, produced from genomic AAV vector DNA templates (identity check). Vectors were stored at −80 °C.

All AAV vectors were produced by the VVF at the University of Zurich.

### 2.9. Transduction of AAV- SaCas9 Vectors

FeLV-A/Glasgow-1-infected CRFK (5.0 × 10^4^ cells/well), FEA (2.5 × 10^4^ cells/well), and PG-4 (5.0 × 10^4^ cells/well) cells were seeded in 24-well tissue culture plates. After 24 h, cells were transduced in triplicates with vT1-5 and v426, a control AAV-DJ/*Sa*Cas9 vector without sgRNA, at an MOI of 10,000. Three wells per cell line were not transduced with the AAV vectors (control cells). On day 3, cells and supernatant were collected from each well.

### 2.10. Statistics

Statistical analyses were performed using GraphPad Prism for Windows, Version 8.4.3 (GraphPad Software, San Diego, CA, USA). Differences among three and more groups were tested for significance using the non-parametric Kruskal–Wallis one-way ANOVA by Ranks (p*_KW_*) for unpaired samples, followed by the Dunn’s Multiple Comparison test (*p*_D_). Percentage NHEJ, determined by TIDE and T7E1, was analyzed using the Spearman rank test for correlation. A *p*-value < 0.05 was considered significant.

## 3. Results

### 3.1. Cell Tropism of AAV Serotypes

**Figure 3:**
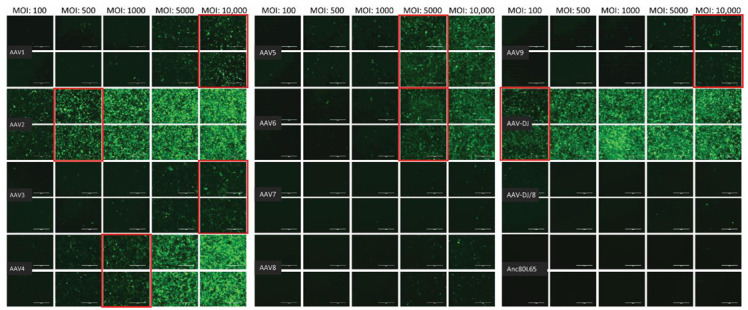
Fluorescent microscopy images of CRFK cells infected with 12 AAV serotypes after three days. Infection of nine natural AAV serotypes (AAV1-9), two AAV hybrid strains (AAV-DJ and AAV-DJ/8), and Anc80L65, all EGFP tagged from the upper left to the lower right, using a multiplicity of infection (MOI) between 100 and 10,000. From the left to the right of each column, the MOI consisted of 100, 500, 1000, 5000, and 10,000, always in duplicates (*N* = 2). Red squares mark the minimal MOI, at which a significant AAV infection could be visualized for each AAV serotype by fluorescence (corresponding to ≥ 50% infection). For AAV7, AAV8, AAV-DJ/8, and Anc80L65, no infection could be observed up to an MOI of 10,000. The bars represent 400 µm.

**Figure 4:**
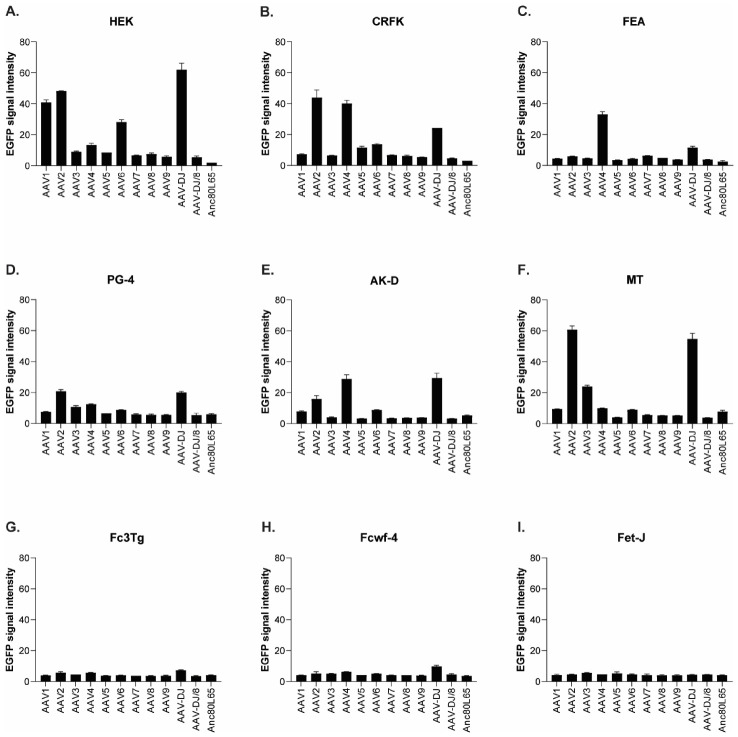
Quantification of the mean fluorescence signal intensity of cells infected with 12 AAV serotypes. (**A**) HEK (MOI of 10,000), (**B**) CRFK (MOI of 10,000), (**C**) FEA (MOI of 10,000), (**D**) PG-4 (MOI of 100,000), (**E**) AK-D (MOI of 100,000), (**F**) MT (MOI of 50,000), (**G**) Fc3Tg (MOI of 50,000), (**H**) Fcwf-4 (MOI of 10,000), and (**I**) Fet-J (MOI of 100,000) cells infected in duplicates (*N* = 2), with 12 AAV serotypes, all EGFP tagged. Mean signal intensity is shown for the highest MOI applied in the respective cell line, ranging from 10,000 to 100,000 on day 3. The signal strength ranges between 0 (black) and 255 (white).

**Figure A2:**
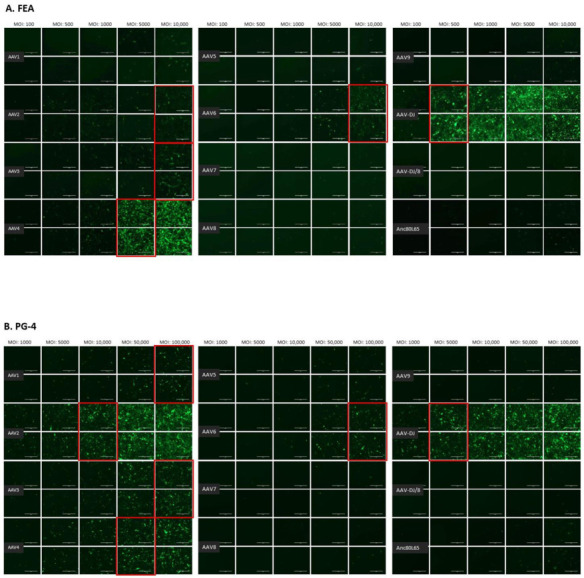

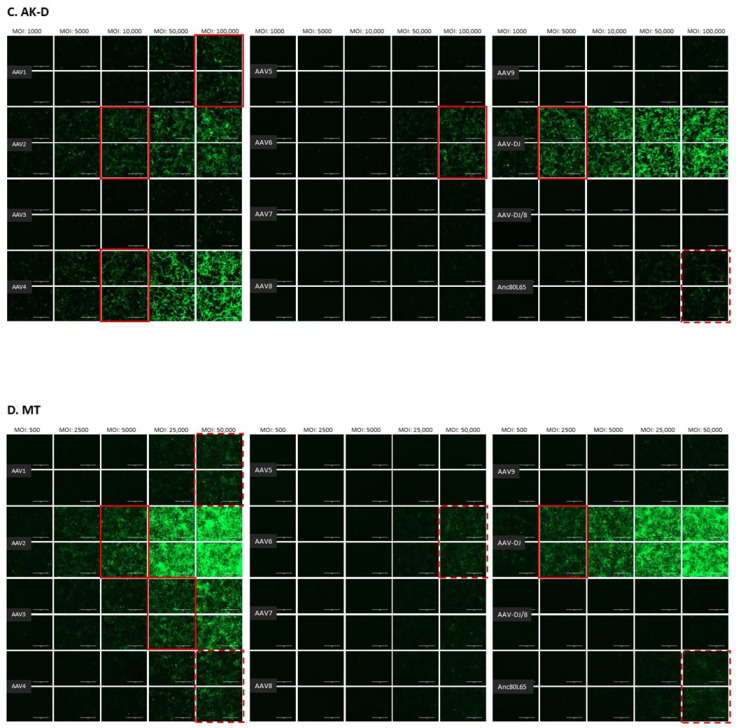

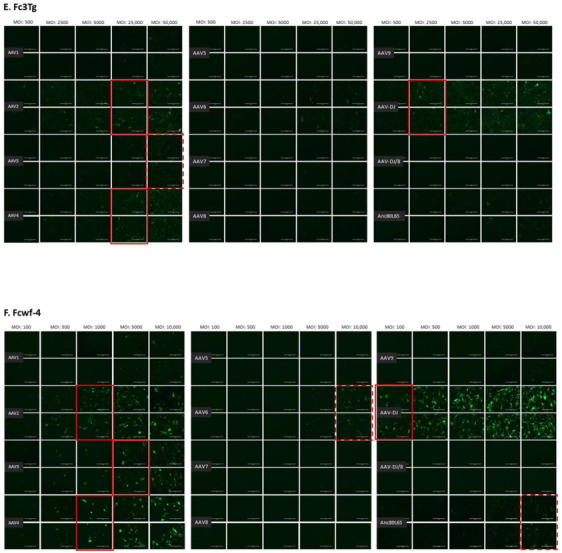

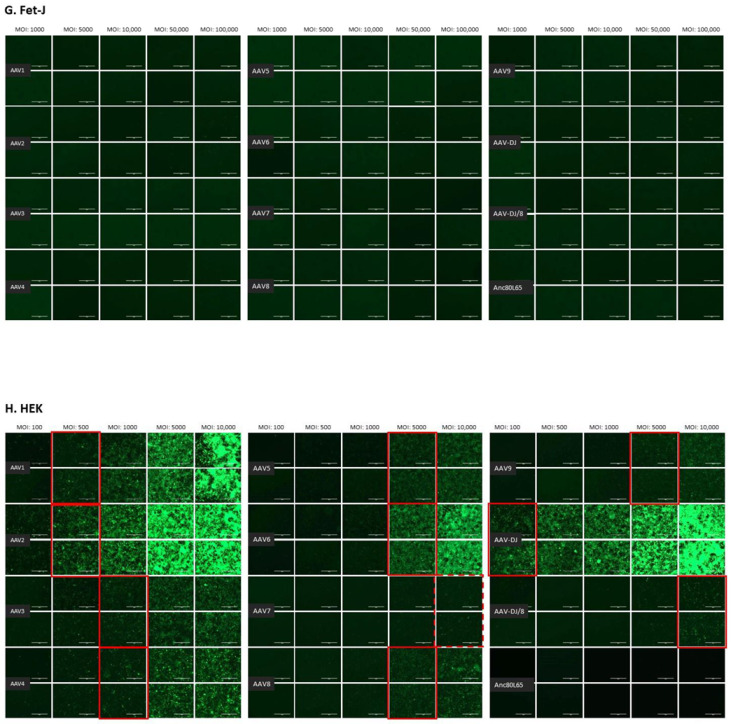
Fluorescent microscopy images of cells infected with 12 AAV serotypes. (**A**) FEA, (**B**) PG-4, (**C**) AK-D, (**D**) MT, (**E**) Fc3Tg, (**F**) Fcwf-4, (**G**) Fet-J, and (**H**) HEK cells infected with 12 AAV serotypes. Infection of nine natural AAV serotypes (AAV1-9), two AAV hybrid strains (AAV-DJ and AAV-DJ/8), and Anc80L65, all EGFP tagged from the upper left to the lower right, using a multiplicity of infection (MOI) between 100 and 100,000 (specific MOI depended on the cell line) after 3 days. Red squares mark the minimal MOI at which a significant AAV infection could be visualized for each AAV serotype by fluorescence (corresponding to ≥50% infection); red dashed squares represent the minimal MOI, at which a EGFP signal was clearly visible, but not reaching 50% infection. The bars represent 400 µm.

The infectivity of 12 EGFP-expressing AAV serotypes (Appendix A, Figure A1) was analyzed by transducing different cell lines, as well as feline PBMCs. We were able to infect most cell lines investigated, with AAV-DJ being by far the most efficient in vitro (Figure 3 shows a representative infection of CRFK cells; the results using other cell lines can be found in Appendix A, Figure A2A–H; Figure 4, and a numeric summary, is given in Table 2). While AAV-DJ was able to infect CRFK and Fcwf-4 cells already at an MOI of 100, the majority of FEA cells were infectable at an MOI of 500. Although all other feline cell lines needed a higher MOI for an efficient infection, AAV-DJ was able to infect all investigated infectable cell lines more efficiently than the other AAV serotypes. While AAV2 and AAV4 also infected several of the cell lines efficiently (Figure 4, Table 2), AAV5, AAV7, AAV8, AAV9, AAV-DJ/8, and Anc80L65 clearly showed less potential for infection in a broad variety of feline cell lines. AAV1, AAV3, and AAV6 showed efficient infection in two or more feline cell lines, thereby revealing an intermediate infection pattern. The T lymphocytic cell line, FetJ, and feline PBMCs could not be infected with the AAV serotypes and the MOIs used in the present study (Figure 4I, Appendix A, Figure A2G, and data not shown). Thus, even at an MOI of 100,000, none of the included AAV serotypes were able to infect FetJ cells or PBMCs, at least not to a level where any clear EGFP-signal was detectable.

When comparing the infectability of cell lines, CRFK and HEK cells were clearly the ones which were most easily infected, followed by Fcwf-4 and FEA. With the exception of FetJ and fPBMCs, all cell lines were infectable by at least some AAV vectors. In general, a dose-dependent expression of EGFP could be observed, with low MOIs showing the least expression and high MOIs showing the highest expression. AAV-DJ clearly showed the highest infectivity of the feline cells tested, followed by AAV2 and AAV4.

### 3.2. FeLV-A/Glasgow-1 Infection of Feline Cell Lines

**Figure 5:**
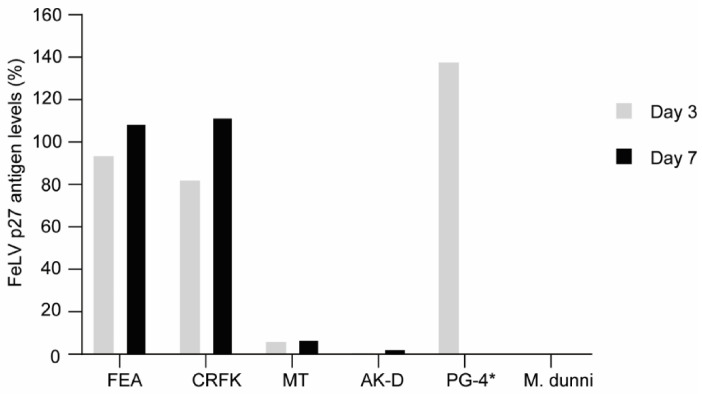
Comparison of FeLV-A/Glasgow-1 infection in various feline cell lines. FeLV p27 antigen levels, as determined by ELISA, after three and seven days. The non-infectable M. dunni cell line was used as a negative control. * Due to the extremely high p27 levels in PG-4 cells, the value depicted only represents day three.

FEA, CRFK, and PG-4 cells were successfully infected with FeLV-A/Glasgow-1 displaying p27 antigen levels > 100% three to seven days after infection. Other feline cell lines, such as MT and AK-D, showed low p27 antigen levels (<8% and <3%, respectively; Figure 5). Thus, all following experiments, to target FeLV provirus using the CRISPR/*Sa*Cas9 system, were performed with FEA, CRFK, and PG-4 cells.

### 3.3. Validation of CRISPR/SaCas9 Targets by Transfection

Lipofectamine transfection of the different CRISPR/*Sa*Cas9 plasmids containing sgRNAs targeting sites within the FeLV provirus resulted in low transfection efficiency, which ranged from 3% (CRFK) to 20–30% (PG-4 and FEA cells). Therefore, a FACS-based cell enrichment assay was performed, where cells successfully transfected with *Sa*Cas9-EGFP-TX were collected, based on a medium to high EGFP signal.

Each target region in the FeLV provirus was amplified with a primer pair specific for exogenous FeLV. Endogenous FeLV (enFeLV) was not detectable in non-infected cell lines using these PCR systems, confirming the specificity of the primers for exogenous FeLV.

**Figure 6:**
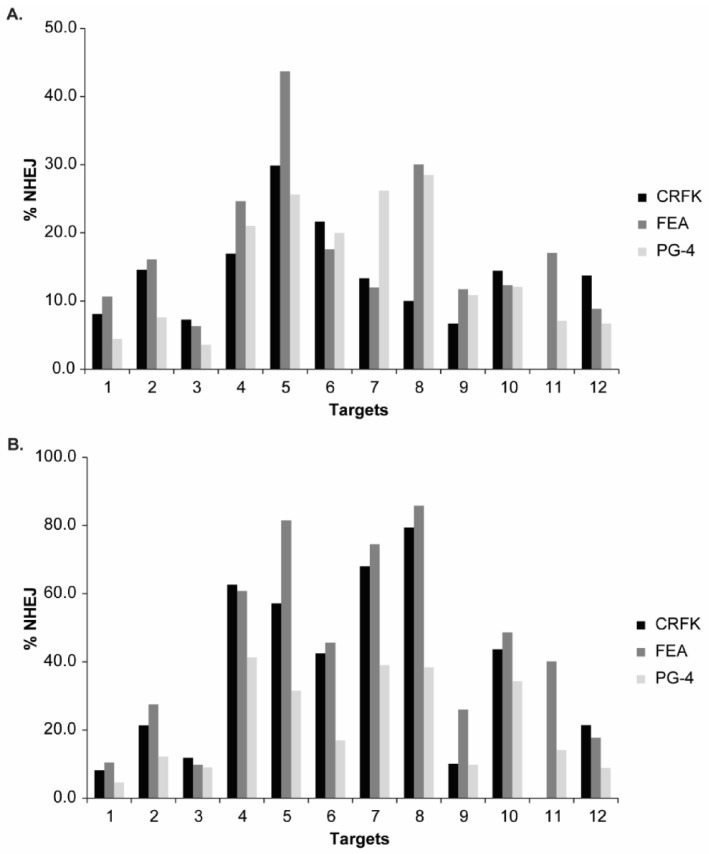
NHEJ frequency in CRFK, FEA, and PG-4 cells using CRISPR/*Sa*Cas9 editing for 12 FeLV targets and analyzed with the T7E1 and TIDE assay. After CRISPR/*Sa*Cas9 transfection and cell sorting, all 12 target regions within FeLV were amplified and analyzed using either the T7E1 assay measured by FA (**A**) or TIDE (**B**). Cleavage frequency, as determined by FA, was calculated into % NHEJ. Three cell lines (CRFK, FEA, and PG-4) are shown for each target and plotted against their % NHEJ (*y*-axes; % NHEJ = 100 × (1 − $\sqrt{1-\frac{\left(a+b\right)}{\left(a+b+c\right)}}$), with *a* and *b* being the cleaved DNA and *c* being the uncut DNA [51]). For target 11, there were not enough CRFK cells for cell sorting available.

**Figure A3:**
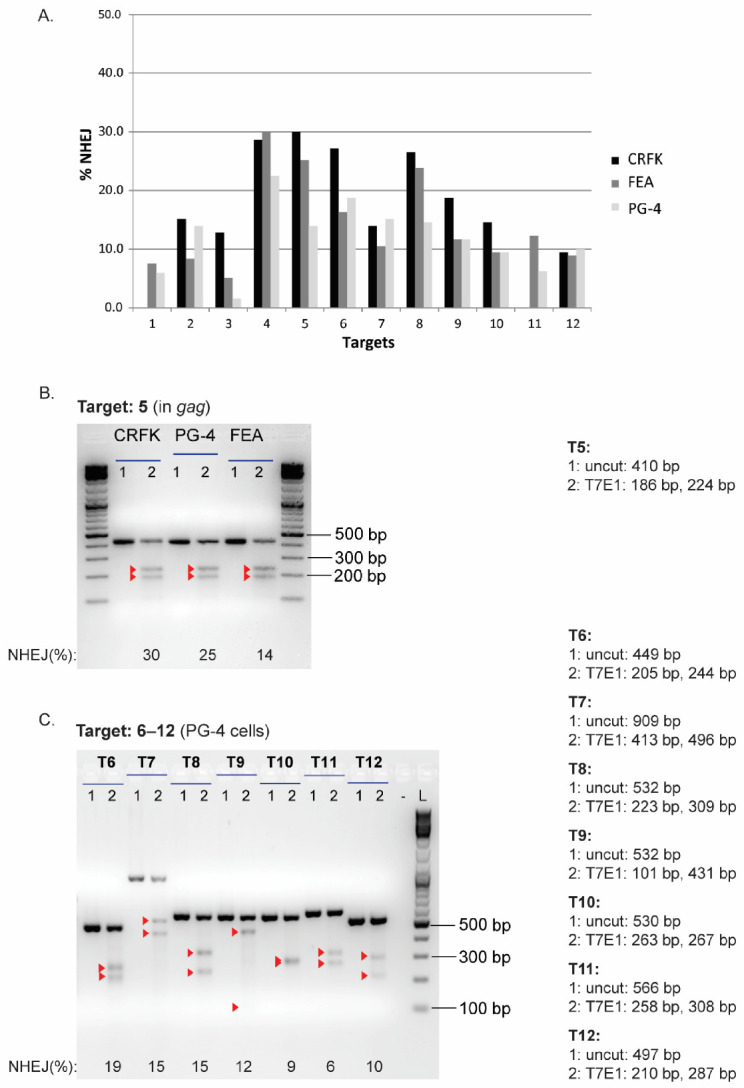
*Sa*Cas9 cleavage efficiency in three cell lines determined by T7E1, visualized on an agarose gel. T7E1 assay showing percentage of NHEJ at the 12 FeLV target sites after transfection of CRFK, FEA, and PG-4 cells and sorting (**A**). Selected results showing *Sa*Cas9 cleavage efficiency at the Target 5 in CRFK, PG-4, and FEA cells (**B**) and Targets 6–12 in PG-4 cells (**C**) visualized after T7E1 cleavage on agarose gels. Red triangles indicate cleaved fragments.

The *Sa*Cas9 cleavage efficiency of the different sgRNAs was visualized after T7E1 cleavage on agarose gels, confirming the successful targeting at all 12 locations in the FeLV provirus (NHEJ frequency and representative results can be found in Appendix A, Figure A3). Additionally, T7E1 cleavage was determined by parallel capillary electrophoresis using a FA. An overview of the calculated NHEJ from the cleavage efficiency, using both the FA and band intensity comparison, is depicted in Figure 6A, Appendix A, Figure A3A, and Table 3. The majority of NHEJ in the sorted cells ranged between 6 and 27%, with targets in the *gag* and *pol* region (T4–T8) being generally higher than in the other regions of the FeLV provirus.

Spearman correlation coefficients were determined to assess the linear relationship between %NHEJ, determined by T7E1 cleavage using a FA and visualization on an agarose gel. In all three cell lines, a linear relationship was found between the two methods for analyzing the T7E1 cleavage efficiency (CRFK: *r* = 0.6182, *p* = 0.0478; FEA: *r* = 0.8671, *p* = 0.0005; PG-4: *r* = 0.8091, *p* = 0.0022).

Additional knowledge about the indel spectrum and relative frequency, after CRISPR/*Sa*Cas9 editing, was gained by TIDE analysis [51]. In general, TIDE analysis revealed a similar pattern, with highest NHEJ in the *gag* and *pol* region (Targets 4–8) and lowest in the LTR (Targets 1–3). An overview of the determined overall efficiency can be found in Figure 6B and Table 3. Generally, higher % NHEJ were calculated by TIDE, in comparison to the T7E1 assay. Targets with low % NHEJ, determined by TIDE (T1, T2, T3, and T9), mostly showed a narrow indel distribution, meaning that indels mainly consisted of a single deletion or insertion (−1 to +1 bp). Targets which seemed more efficiently cleaved tended to show broader mutation patterns with indels of frequently −10 to +3 bp. Interestingly, the broadest indel spectrum was observed in the Target T8: a broad peak was observed between around −10 to +1 bp, with highest indel rate at −2 bp, with around 24% for CRFK and FEA cells.

Due to the promising results found in targets in the *gag* and *pol* region, further evaluation was attempted by the production of gene therapy vectors containing the gene editing construct, as well as a chosen target site.

### 3.4. CRISPR/SaCas9 Activity on the Different FeLV Targets Using AAV-DJ as Gene Therapy Vector

Based on the superior transduction efficiency (Figure 3 and Figure 4, Table 2, and Appendix A, Figure A2A–G), AAV-DJ, an AAV hybrid strain, was selected as the delivery vector for *Sa*Cas9-sgRNA. In order to ensure the highest possible transduction rates, an MOI of 10,000 was chosen for all the transduction experiments. FeLV-A/Glasgow-1-infected CRFK, FEA, and PG-4 cells were transduced with AAV-CRISPR/*Sa*Cas9 vectors either with a FeLV-specific sgRNA-sequence (Targets 4, 5, 6, 8, and 10) or without (control AAV-CRISPR/*Sa*Cas9 without target sequence: v426, Appendix A, Figure A1B).

**Figure 7:**
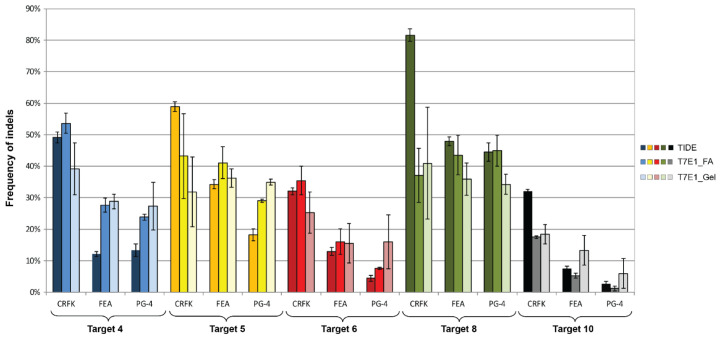
Comparison of CRISPR/*Sa*Cas9 activity leading to indels after transduction. After CRISPR/*Sa*Cas9 transduction using the AAV-DJ vectors vT4 (Target 4, blue), vT5 (Target 5, yellow), vT6 (Target 6, red), vT8 (Target 8, green), and vT10 (Target 10, grey); NHEJ events were compared using TIDE and the T7E1 assays. T7E1 efficiency was determined by parallel capillary electrophoresis using a fragment analyzer (FA) and band intensity comparison on an agarose gel. Transductions were performed on CRFK, FEA, and PG-4 cells. Data represent the mean of three biological replicates ± standard deviation.

**Figure A4:**
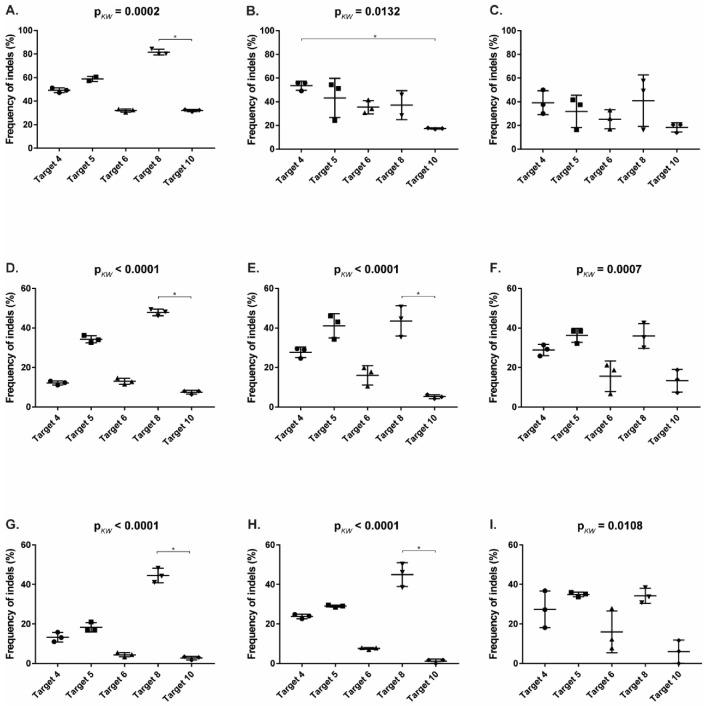
Comparison of CRISPR/*Sa*Cas9 activity leading to indels after transduction. After CRISPR/*Sa*Cas9 transduction using the AAV-DJ vectors vT4 (Target 4, vKHH1), vT5 (Target 5, vKHH3), vT6 (Target 6, vKHH4), vT8 (Target 8, vKHH2), and vT10 (Target 10, vKHH5), frequency of indels (%) were compared using TIDE (**A**,**D**,**G**) and the T7E1 assays. T7E1 efficiency was determined by parallel capillary electrophoresis using a fragment analyzer (FA, graphs (**B**,**E**,**H**)) and band intensity comparison on an agarose gel (**C**,**F**,**I**). Transductions were performed on CRFK (**A**–**C**), FEA (**D**–**F**), and PG-4 cells (**G**–**I**). Frequency of indels (%) was tested for significant differences by Kruskal–Wallis, one-way ANOVA by ranks (p*_KW_* as indicated), and subsequently by Dunn’s post-test: * = *p* < 0.05. The data are shown as scatter dot plots. The horizontal line represents the mean with SD.

**Figure A5:**
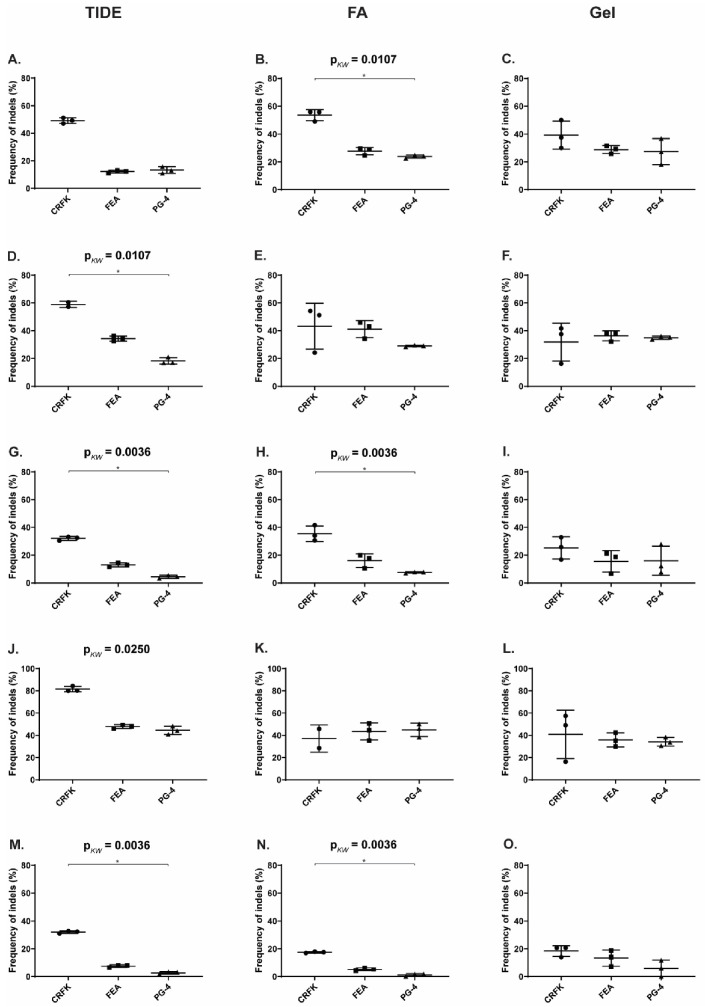
Comparison of CRISPR/*Sa*Cas9 activity between cell lines. After CRISPR/*Sa*Cas9 transduction using the AAV-DJ vectors, the frequency of indels (%) was compared between the cell lines. Each raw corresponds to one Target: graphs (**A**–**C**) to Target 4 (vKHH1); (**D**–**F**) to Target 5 (vKHH3); (**G**–**I**) to Target 6 (vKHH4); (**J**–**L**) to Target 8 (vKHH2); and (**M**–**O**) to Target 10 (vKHH5). The graphs (**A**,**D**,**G**,**J**,**M**) represent results determined by TIDE; (**B**,**E**,**H**,**K**,**N**) by T7E1 using the fragment analyzer (FA); (**C**,**F**,**I**,**L**,**O**) by T7E1 quantifying signal intensity on a gel. Frequency of indels (%) was tested for significant differences by Kruskal–Wallis, one-way ANOVA by ranks (p*_KW_* as indicated), and subsequently by Dunn’s post-test: * = *p* < 0.05. The data are shown as scatter dot plots. The horizontal line represents the mean with SD.

The CRISPR/*Sa*Cas9 activity, on the various target sites, was evaluated using the T7E1 mismatch detection system and the TIDE analysis (Figure 7 and Table 4). The TIDE analysis resulted in less variations within the triplicates of a target, compared to the T7E1 analysis by FA or on a gel (Figure 7, Table 4, details in Appendix A; Figure A4 and Figure A5).

Using the TIDE analysis, the frequency of indels varied between 2.7% (for T10 in PG-4) and 81.6% (T8 in CRFK), by FA between 1.2% (for T10 in PG-4) and 53.6% (T4 in CRFK) and by gel between 6% (for T10 in PG-4) and 41% (T8 in CRFK), confirming the successful introduction of indels (Table 4).

Statistically significant, higher indel frequency was found in Target 8, compared to Target 10 for the TIDE analysis, in all three cell lines and in the T7E1 analysis by FA in FEA and PG-4 cells (Appendix A, Figure A4). With the exception of the TIDE analysis of Target 4 in FEA cells, Targets 6 and 10 always showed the lowest frequency of indels in all analyses.

A comparison between the frequency of indels between the different cell lines within one target showed a tendency of a higher percentage in CRFK cells, compared to FEA and PG-4 cells (Appendix A, Figure A5). Statistically significant differences between CRFK and PG-4 cells were observed for the TIDE analysis for Targets 5, 6, and 10, as well as for the T7E1 analysis by FA for Targets 4, 6, and 10.

Spearman correlation coefficients were determined to assess the linear relationship between %NHEJ, revealed by T7E1 cleavage using a FA and visualization on an agarose gel. A linear relationship of *r* = 0.832 with *p* < 0.0001 was calculated. A significant correlation could also be shown between the TIDE and T7E1, analyzed by FA, as well as between the TIDE and T7E1, analyzed by gel: *r* = 0.873 and *p* < 0.0001; *r* = 0.721 and *p* < 0.0001, respectively.

### 3.5. Reduction of FeLV Antigen in Different Cell Lines by AAV Transduction

**Figure 8:**
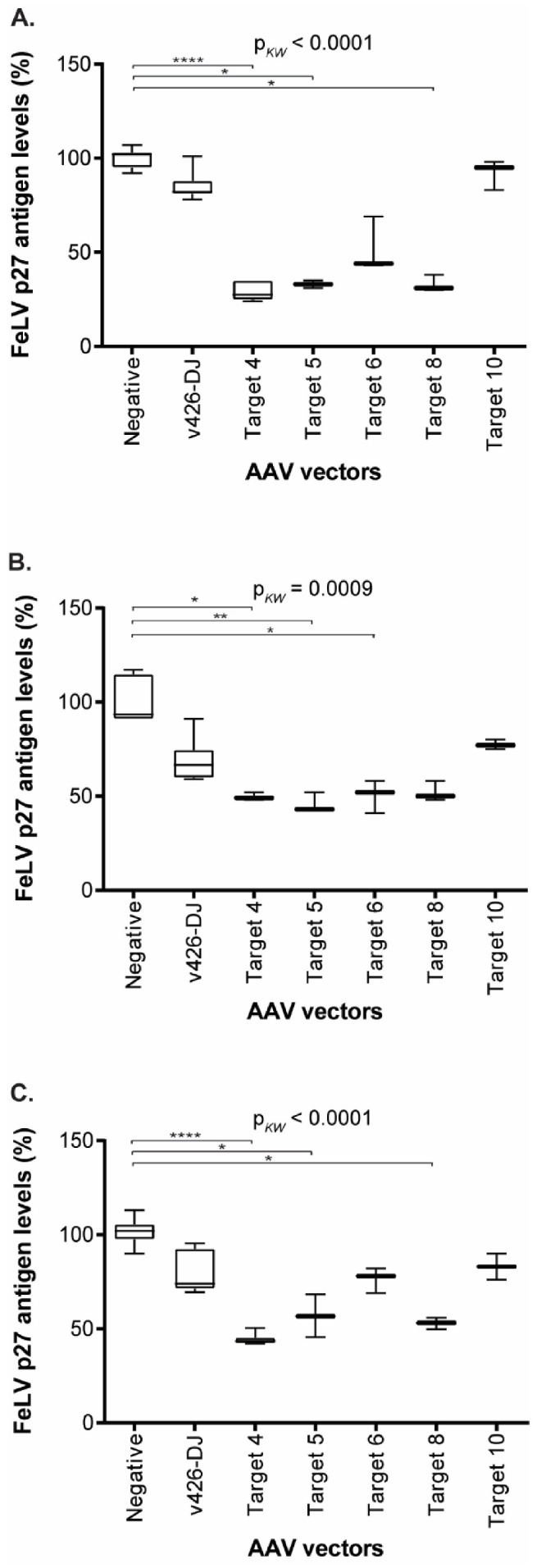
FeLV p27 antigen levels, after transduction in supernatant of CRFK (**A**), FEA (**B**), and PG-4 (**C**) cells. Comparison of FeLV p27 antigen levels after transduction with the AAV-DJ vector without target (v426), Target 4 (vT4), Target 5 (vT5), Target 6 (vT6), Target 8 (vT8), Target 10 (vT10), and non-transduced cells (negative). Since several individual experiments were compared, ultimate normalization of p27 values was performed to the p27 means of the non-transduced control cells (equaling 100%). FeLV p27 antigen levels were tested for significant differences by Kruskal–Wallis one-way ANOVA by ranks (p*_KW_* as indicated) and subsequently by Dunn’s post-test: * = *p* < 0.05; ** = *p* < 0.01; **** = *p* < 0.0001. The data are shown as box plots; the boxes extend from the 25th to 75th percentiles. The horizontal line represents the median, and the whiskers extend from the smallest to the largest value.

The transduction of the three cell lines with the gene therapy vectors vT4, vT5, vT6, VT8, and vT10 resulted in a significant reduction in FeLV p27 antigen (Figure 8). Gene therapy vectors vT4 and vT5 showed a statistically significant reduction in p27 antigen, by up to 71%, compared to untreated cells in all three cell lines (vT4: CRFK: *p* < 0.0001; FEA: *p* = 0.0306; PG-4: *p* < 0.0001; vT5: CRFK: *p* = 0.0245; FEA: *p* = 0.0094; PG-4: *p* = 0.0373). Additionally, FeLV p27 antigen levels were significantly reduced by using vT8, leading to a reduction of 67% in CRFK and 47% in PG-4 cells (*p* = 0.019 and *p* = 0.0228, respectively), whereas vT6 showed only a statistically significant reduction in p27 antigen levels in FEA cells (p27 antigen reduction of 50%, *p* = 0.0459, Figure 8). Target 10, which directs *Sa*Cas9 to a site in the *env*, showed the least reduction in p27 antigen production using all three cell lines (Figure 8).

## 4. Discussion

The persistence of retroviral reservoirs in the cellular genome is a major obstacle to curing retroviral infections in animals, as well as in humans. Recent technologies have provided the means to target retroviruses in their most hidden state—as a provirus. Thus far, retroviral treatments generally focused on the virus, thereby allowing the provirus, hidden in the host, to thrive. With the discovery of CRISPR, a highly specific, easily programmable gene editing technology, the goal of deleting or deactivating the provirus itself has become a possibility. In contrast to HIV (a lentivirus), the sequence of FeLV (a gammaretrovirus) is highly conserved throughout infection [58]. Because the efficacy of the CRISPR/Cas9 system is largely dependent on sgRNA matching the target viral DNA, its application should, thus, be even more effective for FeLV. While we recognize that differences exist between FeLV gammaretrovirus infection in cats and HIV lentivirus infection in humans, this natural animal model may still yield proof-of-concept results, in support of the efficiency of using the CRISPR/Cas9 system to target retroviral provirus sequences and reduce proviral loads in vitro and in the future in vivo in an outbred animal species. In addition, after natural FeLV infection, most naïve cats can mount successful immune responses. These cats are regressively infected with either no or only transient viremia and subsequent suppression of virus replication and the absence of development of FeLV-associated diseases. Thus, it may not be necessary to target and destroy every provirus in progressively infected cats. Rather, by reducing (pro)virus levels, the immune system of the cat may be able to take the lead and suppress the remaining virus.

Among the various strategies for the transport of bioreactive molecules, AAV has stood out, due to its broad variety of available vectors, safety, and low induction of immunologic reactions. A constantly increasing amount of AAV serotypes has been discovered, as well as designed, addressing various features. In the first step, AAV vectors needed to be found to specifically target cells containing FeLV provirus. Thus, in this study, we systematically tested the infectivity of various AAVs in different feline cell lines (as a model for different feline tissues) for their ultimate application as suitable gene therapy vectors. The receptor of FeLV-A, fTHTR1, the thiamine transport protein 1, was found in a broad variety of tissues, with highest levels in sites of FeLV entry and shedding [59,60]. Consequently, FeLV has a very broad cell target spectrum and, thus, a vector that targets many different feline tissues was sought. Various AAV vectors were tested in vitro for a matching tropism. Differences between cell lines, as well as AAV serotypes, were obvious. AAV-DJ, a hybrid strain, was clearly superior in infecting a broad range of cell lines. AAV-DJ had been designed using a DNA family shuffling technology, with the goal of improving the desired qualities of multiple AAV isolates [61]. Similar to earlier results in human cell lines [61], AAV-DJ was superior in its in vitro infectivity to all tested AAV serotypes in feline cell lines. With the exceptions of FetJ and fPBMCs, AAV-DJ could infect all tested cell lines. Similar findings had already been described for human monocytes and dendritic cells where other serotypes, namely, AAV-1 and AAV-6, outperformed AAV-DJ [61]. Thus, virus infectivity in vitro might not necessarily reflect the FeLV tropism in vivo completely, since AAV-DJ lacked the ability to infect T-lymphocytes and fPBMCs. Therefore, additional AAV serotypes, or other vectors, potentially need to be screened in further studies to gain access to all target cells of FeLV.

When potential target sites were chosen for this study, each region in the FeLV, corresponding to the LTR, *pol*, *gag*, and *env*, was addressed. These sites were chosen, according to their suitability, based on in silico algorithm prediction and the presence of the *Sa*Cas9 PAM site. Moreover, they were checked for potential sequence identity to the feline genome. None of the 12 targets revealed sequence identity within the coding region to the cat’s genome. Two of the targets (Target 2 and 10) did not reveal sequence identity to the cat’s genome at all (including non-coding sequences). In contrast, Target 7, located in the *pol* region of FeLV, showed sequence similarity with enFeLV-like sequences. Domestic cats, and related small felids (genus *Felis*), harbor enFeLV in their genome: footprints of ancient retroviral infections [62,63]. Endogenous retroviruses are a highly diverse group of elements, with a gene organization closely related to that of exogenous retroviruses [64]. Although the transcription and translation of enFeLV has been demonstrated in healthy cats and in feline cell lines, enFeLV are typically incapable of giving rise to infectious virus particles [65,66]. An association between enFeLV loads and FeLV replication was demonstrated in domestic cats, where only five of ten cats with high enFeLV loads, but nine of ten cats with low enFeLV loads, developed progressive FeLV infection [67]. The later observation was subsequently substantiated, when a significant association between enFeLV loads and the infection outcome was found in another experimental study: cats that developed regressive infection had significantly higher enFeLV loads than cats that developed progressive FeLV infection [68].

Not only in domestic cats, but also in pumas, has it been shown that endogenous viral elements negatively correlate with FeLV susceptibility [63]. It was hypothesized that, due to the absence of enFeLV in felids outside the genus *Felis*, more severe disease consequences might, therefore, be observed [63]. *Puma concolor* samples lacking enFeLV were shown to be more permissive to FeLV infection than domestic cat samples [63]. In addition, enFeLV-LTR elements seem to negatively correlate with exogenous FeLV replication [63]. If target sequences for CRISPR/*Sa*Cas9 on the exogenous FeLV are identical to enFeLV sequences, enFeLV also becomes a target. This effect on the enFeLV copy numbers might have implications not yet fully realized. In the present in vitro study, only Target 7 had 100% identity with enFeLV sequences.

Overall, twelve target sites, distributed within the FeLV provirus, were compared for their rates of DNA editing by CRISPR/*Sa*Cas9 using transfection. The highest efficacy in indel creation was found in the targets localized in the *gag* and *pol* (Targets 4, 5, 6, and 8). Thus, in a follow-up experiment, five targets, including the four mentioned above, resulting in highest indel production and the most promising target site in the *env*, T10, were further investigated in a transduction study. Again, the highest indel efficiency was found in regions targeting the *gag* and *pol*.

In addition to the anticipated specific editing of target sites, undesired off-target nucleotide changes often represent obstacles for successful applications. In treatments, such as the targeting of the FeLV provirus, in particular, which is integrated in the genome all over the organism, off-site targeting needs to be investigated closely. Investigations of *Sa*Cas9, the enzyme used in the present study, revealed double-strand breaks at a subset of off-target sites, previously identified for the widely applied *Sp*Cas9, with significantly lower read counts [69]. In addition, recent publications have described new editing approaches, with improved specificity of base editors [70,71]. Thus, potential future steps towards in vivo experiments should closely investigate these risks, in order to find a safe treatment option.

DNA editing could be shown by T7E1 and TIDE, at all chosen target sites, with the highest % NHEJ in the highly conserved *gag* and *pol* regions. Five promising candidates were further investigated during a transduction experiment, where AAV-DJ gene therapy vectors were designed, containing the CRISPR/*Sa*Cas9, as well as the target information. Again, successful gene editing was confirmed by T7E1 and TIDE analyses. FeLV p27 antigen was quantified, in order to determine the effect of these indels. A significant reduction in all three cell lines, for the two gene therapy vectors vT4 and vT5, both targeting the FeLV *gag*, was found. While Target 5 is localized in the p27 core protein, Target 4 and Target 6 are both localized in p15.

However, a significant provirus reduction using qPCR, targeting either *env* or *gag*, could only be shown for Target 8 in CRFK cells (Supplementary Figure S1 and data not shown, respectively). It needs to be mentioned that primers and probes of both qPCR assays did not cover any of the specific target sites. An earlier study by Chakrabarti et al. showed that the most common CRISPR/Cas9 indel mutations are 1 bp insertions or deletions (with 44% and 26%, respectively), leading to frameshifts [72]. Thus, this indicates that the FeLV provirus sequence is likely still present in the genome. A reduction of provirus, by qPCR quantification, might not have been observed, even if nearby sequences were mutated, leading to provirus numbers in the same range as our controls. In addition, some cells might not be transduced by AAV, thus still producing intact provirus and continuing with the production of infectious FeLV, resulting in a dynamic process. In addition, all measurements were performed on day 3; perhaps a more pronounced effect could have been seen at a later time point. It would be highly interesting to analyze induced mutations and viral progenies. Studying their infectivity efficiency and tropism should be investigated in a follow-up study.

Differences between the targeted and the original provirus might only lay in their ability to produce intact antigens, which are necessary to produce infectious virus particles. Insertions or deletions of 1–2 bp can lead to frameshifts, which might impair the entire infection cycle. Experiments in all three cell lines clearly showed the successful applicability of CRISPR/*Sa*Cas9-mediated gene editing leading to a reduction of FeLV p27 antigen levels. Thus far, only single targets have been investigated. However, combinations of multiple targets might even provide better results. Thus, future studies should not only include the most promising candidates in the *gag* and *pol* regions, but also include targets in the LTR. A combination of targets further apart might increase the chances for excision between two different sgRNAs and, thus, result in the loss of a bigger FeLV region. Studies using *Sp*Cas9 with multiplex sgRNAs in vitro, as well as in vivo (mouse model), to excise HIV-1 provirus showed great promise, not only in enhancing cleavage efficiency but also in preventing potential HIV escape, due to its high mutation rate [73].

Several established methods provide the means to determine the extent and efficiency of CRISPR/*Sa*Cas9-induced gene editing. High-throughput sequencing, with next-generation sequencing (NGS) as a gold standard, can analyze an entire mutation spectrum. Despite high error rates in GC- and AT-rich regions and homopolymer stretches, in general, its error rate is low at <0.4% [74] and improving constantly with newly developed NGS platforms. By producing a huge amount of information and, alongside this, the need for sophisticated data analysis, the costs remain significant, despite recent reductions. Alternative methods have evolved, which provide a simpler and cheaper approach to measure the efficiency and extent of genome editing events.

T7E1 is a structure-selective enzyme that is widely used to detect DNA mismatches. Its straight-forward and cost-effective approach provides semi-quantitative information on the presence of heteroduplexes representing nucleotide mismatches, as a result of NHEJ [50]. Although this method provides information about the amount of mutated sequences, their nature and diversity remain unknown.

Alternatively, the TIDE algorithm is a fast and cost-effective approach, accurately quantifying the editing efficacy and predominant types of indels [52]. By analyzing and comparing Sanger sequence traces, generated from convoluted WT and edited samples, the TIDE software identifies and quantifies induced mutations. Indel estimates with TIDE have been shown to be typically congruent with those from NGS; however, the sensitivity of TIDE can only identify indels up to about 1–2% [51].

Apart from NGS, T7E1, and TIDE, other site-specific nuclease validation systems also exist, such as the Indel Detection by Amplicon Analysis (IDAA) assay [75]. This fast, sensitive, and simple method is based on tri-primer amplicon labelling and DNA capillary electrophoresis detection. Comparisons of TIDE and IDAA revealed similar indel frequency results; however, their numbers were about 10–20% lower than targeted NGS [51]. Previous studies showed that using the NGS approach for assessing editing efficiencies in cell pools can accurately portray editing events [51]. Compared to TIDE and T7E1, NGS seems to be more reliable and robust over a broad spectrum of indel sizes; however, due to its labor- and cost-intensive features, it is not always feasible to use this method.

Due to cost effectiveness, as well as efficiency, the CRISPR/*Sa*Cas9 target sites, investigated in the present study, were analyzed by T7E1 assay and compared to the TIDE algorithm output. When comparing the data obtained by T7E1, measured on a FA or by gel and TIDE, a significant correlation was found. However, although general trends, such as the most efficiently cleaved target, were consistent in the different techniques, the specific % NHEJ events were not. The reasons for this are manifold and can partially be explained by how the different assays work. T7E1 is an endonuclease, which cleaves wherever mismatches in the DNA causes heteroduplexes to form. It easily detects indel variants; however, single nucleotide polymorphisms are poorly recognized [52,76]. Thus, samples with greater indel size heterogeneity, based on TIDE analysis, more closely reflect the overall editing efficiency by T7E1. In addition, a prerequisite for this technique is the presence of different sequences, resulting in a plateau, which has been described at around 40–50% [51,76]. The results found in our study might suffer from such a plateau situation, in some cases. Thus, even with very high NHEJ, a low and limited detection range is expected, which might be even more pronounced, in the case of a very narrow mutation range. Depending on the method of evaluation, another bias needs to be addressed. The choosing of the band area, within an agarose gel after T7E1 digestion, might influence the final measurements of NHEJ. In order to provide a second evaluation of the efficiency of T7E1 digestion, this experiment was also analyzed on a FA. In addition, T7E1 and TIDE are both methods which are PCR-based and, therefore, have additional limitations. These include, but are not limited to, not being able to detect large mutations, which tamper with the binding sites of the primers, as well as the possibility of a bias for smaller amplicons. Despite all these limiting features, a general trend was obvious pointing towards the highest % NHEJ in the highly conserved *gag* and *pol* regions. In addition, TIDE analysis revealed higher amounts of deletions, compared to insertions for all the sites investigated during transduction.

Cats infected with FeLV can undergo various outcomes. Among them, viremic sick cats showed the highest FeLV provirus and viral loads, whereas regressively infected cats showed significantly lower levels [13]. Unlike HIV and the feline immunodeficiency virus (FIV; the lentivirus of the cat), where the virus resides mainly in peripheral CD4+ T cells, FeLV has been found in a broad variety of cell types. However, viral RNA and provirus loads vary significantly between different tissues, with the highest loads in viremic sick cats in the lymphoid tissues, intestinal tract, and salivary glands [13]. Provirus tissue loads were found to reach levels of several copies per cell [13], clearly stating that any attempt targeting this provirus also needs to access all these different cell types. Although AAV-DJ was clearly superior in infecting different feline cell lines in vitro, when compared to wild-type AAV serotypes, AAV-DJ/8, and Anc80L65, in vivo experiments using AAV-DJ will be necessary to corroborate these results. Thus, if no gene therapy vector can be found infecting most tissues in vivo, studies might need be directed to the three main areas of FeLV infection: the lymphoid tissues, the intestinal tract, and the salivary glands. It will be interesting to see whether this system, thus far successfully applied in vitro, will also succeed in vivo.

Previous studies using CRISPR editing in FIV-infected feline T lymphocyte cell lines discussed the possibility of overwhelming the functionality of the gene editing method by using an acute FIV infection strategy versus targeting latently infected cells [77]. The latent reservoir in peripheral CD4+T cells, during the asymptomatic phase of FIV-C infection, was found to be approximately one in 10^3^ cells, of which only one in 10^2^ was also replication competent, resulting in an overall infection rate of around one in 10^5^ cells [78]. Thus, it was stated that the lentiviral gene editing strategy might be more effective in latently FIV-infected cats [77]. Due to the biology of FeLV infection and the different outcomes, that depend on the balance of the virus and the immune system of the cat [79], we hypothesize that even incomplete provirus destruction, accompanied with a reduction of viral replication, might provide the immune system with the means to overcome progressive infection and enable the cat to reach a regressive infection outcome. Since such a change in FeLV infection outcome results in significant differences of the cat’s survival expectancy, as well as non-infectivity to co-housed cats, a regressive infection outcome would already be highly desirable. Of course, complete elimination of the FeLV provirus would be even more attractive, but its achievability remains unclear.

Consistent with our aim to cure cats from progressive FeLV infection, and while our experiments were underway, CRISPR/Cas9 strategies have also been applied in various studies, with the goal of achieving a permanent cure for HIV/AIDS. Thus, the *Sp*Cas9 system has been successfully applied in the disruption or excision of HIV itself, as well as its co-receptors CCR5 and CXCR4 [80,81,82,83]. Enhanced HIV-1 excision in cultured cells could be obtained by a combination of sgRNAs targeting LTRs and the viral structural genes in the *Sp*Cas9 system [84]. A proof-of-concept study demonstrated, also in vivo, excision of HIV-1 DNA by CRISPR/*Sa*Cas9 upon delivery, by tail vain infection of transgenic mice or retro-orbital inoculation in transgenic rats, using an AAV9 gene therapy vector [85]. Thus, future studies using *Sa*Cas9 to target FeLV provirus might benefit from some of the experience gained in the HIV studies. Nonetheless, some differences exist between FeLV and HIV that will make the combat against FeLV more challenging, such as the broad tissue tropisms of FeLV. In contrast, the natural ability of cats to overcome viremia might come in helpful. If we are able to destroy even only a part of the provirus, this might be sufficient to lower the virus burden to a level where the cat’s immune system can play an active role and gain control over the virus. Therefore, complementary strategies, such as boosting innate immunity using oligonucleotide-mediated immune system stimulation, should also be considered when targeting the FeLV provirus [86]. Furthermore, FeLV is rather conserved genetically; in contrast to HIV, it barely mutates. Whereas cases of mutant escapes have been described in HIV; such sequence changes were seldomly found in cats [58]. Ultimately, a lot of evidence points towards a successful application of *Sa*Cas9 in the fight against progressive FeLV infection.

## 5. Conclusions

Curative treatments of many infectious diseases are nonexistent. Thus, new strategies, such as the CRISPR/Cas9 system, need to be explored. Retroviral infections are generally difficult to cure, since the provirus hides integrated within the host’s genome. Provirus destruction does not only depend on highly specific targeting but also on the optimal delivery of bioreactive molecules. To achieve this goal, AAVs, as gene delivery vectors, have been investigated in vitro. The obtained information on tropism, and the efficiency of infection within feline tissues, will be beneficial for future research with a focus on gene therapy.

The present study focused on the application of the CRISPR/*Sa*Cas9 gene editing tool in targeting FeLV-infected feline cell lines. Efficient provirus cleavage could be observed first in transfection and afterwards in transduction experiments, with the highest indel efficiency in the *gag* and *pol* region of FeLV. This study shows a proof of principle in the application of CRISPR/*Sa*Cas9-mediated gene editing targeting FeLV provirus in vitro. The levels of gene editing are highly encouraging, and therefore, this study is a good starting point, and an important basis, for future in vivo experiments in cats infected with FeLV.

## Figures and Tables

**Table 1:** Selected *Sa*Cas9 target sites, PCR primers and conditions.

Target	Direction ^1^	Sequence	Amplification Primers	Annealing	PCR Size	T7E1 Cut
Target 1	as	TTATATCTCATGGGAACATAC	907_F: CCCCTACCCCAAAATTTAGCC	64 °C	456 bp	386 bp
		GTATGTTCCCATGAGATATAA	1710_R: GTCTTCCTCGGCGATGAGAC			70 bp
Target 2	s	GAGGCCAAGAACAGTTAAACC	907_F: CCCCTACCCCAAAATTTAGCC	64 °C	456 bp	296 bp
		GGTTTAACTGTTCTTGGCCTC	1710_R: GTCTTCCTCGGCGATGAGAC			160 bp
Target 3	s	TCTCCAGGCTCCCCAGTTGAC	907_F: CCCCTACCCCAAAATTTAGCC	64 °C	456 bp	229 bp
		GTCAACTGGGGAGCCTGGAGA	1710_R: GTCTTCCTCGGCGATGAGAC			227 bp
Target 4	as	TGCGGCGAAAGAGGTTGAGGG	1229_F: GAAACCGTCATGGGCCAAAC	65 °C	714 bp	373 bp
		CCCTCAACCTCTTTCGCCGCA	1366_R: AGTTAGGGCCACTGGATCTT			341 bp
Target 5	s	CCTGACAGGCGAAGAAAGGCA	1365_F: CAACAACCGACCCCAGTATT	65 °C	410 bp	224 bp
		TGCCTTTCTTCGCCTGTCAGG	1691_R: CAACCTGCTTTACCTGTGCC			186 bp
Target 6	as	TAGTAATGTAAGGAACTTGGT	1229_F: GAAACCGTCATGGGCCAAAC	65 °C	449 bp	244 bp
		ACCAAGTTCCTTACATTACTA	1230_R: GAGGAAGGATCAGGCGGTAAC			205 bp
Target 7	s	TTATACCGGGTACGTAACACG	1248_F: CCGGAACACATTGGGAAG	61 °C	909 bp	496 bp
		CGTGTTACGTACCCGGTATAA	1251_R: CCCACCAGAAACGCTAAG			413 bp
Target 8	as	GTACCAAGGGTGAGACGGCGG	1236_F: GGTATGGGAATGGCTCATTGTC	61 °C	532 bp	223 bp
		CCGCCGTCTCACCCTTGGTAC	1240_R: GGTACCTTACCCTGAAATCG			309 bp
Target 9	s	TATGCCCCATGAAGCGTACCA	1236_F: GGTATGGGAATGGCTCATTGTC	61 °C	532 bp	431 bp
		TGGTACGCTTCATGGGGCATA	1240_R: GGTACCTTACCCTGAAATCG			101 bp
Target 10	s	ATTTAGTCCCCAGAAAAAGGG	1543_F: CCAACAGGGATGGTTTGAAG	61 °C	530 bp	267 bp
		CCCTTTTTCTGGGGACTAAAT	1544_R: CGGAAGGTCGAACTCTGGTC			263 bp
Target 11	as	GGGACACCGTGAATAAAGCGA	1662_F: CAAAGGGAACACATTGTGGA	61 °C	566 bp	308 bp
		TCGCTTTATTCACGGTGTCCC	1668_R: ATAGGGTGGTCGAGAAACCA			258 bp
Target 12	s	GGCTCTGTGCCGCATTGAAAG	1666_F: AGCTGTCAGGTTCCGAAGAG	62 °C	497 bp	287 bp
		CTTTCAATGCGGCACAGAGCC	1669_R: GGCCGAAGAGGAGAATTAGG			210 bp

^1^ Direction of the target: s: sense; as: anti-sense.

**Table 2:** Summary of in vitro infectivities of various AAV serotypes in different cell lines and fPBMCs depicted as multiplicity of infection (MOI) ^a^.

	HEK	CRFK	FEA	PG-4	AK-D	MT	Fc3Tg	Fcwf-4	FetJ	fPBMC
AAV1	**500**	10,000	>10,000	100,000	100,000	≥50,000	>50,000	>10,000	>100,000	>100,000
AAV2	**500**	**500**	10,000	10,000	10,000	5000	25,000	**1000**	>100,000	>100,000
AAV3	**1000**	10,000	10,000	100,000	>100,000	25,000	≥50,000	5000	>100,000	>100,000
AAV4	**1000**	**1000**	5000	50,000	10,000	≥50,000	25,000	**1000**	>100,000	>100,000
AAV5	5000	5000	>10,000	>100,000	>100,000	>50,000	>50,000	>10,000	>100,000	>100,000
AAV6	5000	5000	10,000	100,000	100,000	≥50,000	> 50,000	≥10,000	>100,000	>100,000
AAV7	≥10,000	>10,000	>10,000	>100,000	>100,000	>50,000	>50,000	>10,000	>100,000	>100,000
AAV8	5000	>10,000	>10,000	>100,000	>100,000	>50,000	>50,000	>10,000	>100,000	>100,000
AAV9	5000	10,000	>10,000	>100,000	>100,000	>50,000	>50,000	>10,000	>100,000	>100,000
AAV-DJ	**100**	**100**	**500**	**5000**	**5000**	**2500**	**2500**	**100**	>100,000	>100,000
AAV-DJ/8	10,000	>10,000	>10,000	>100,000	>100,000	>50,000	>50,000	>10,000	>100,000	>100,000
Anc80L65	50,000	≥50,000	100,000	>100,000	≥100,000	≥50,000	>50,000	≥10,000	>100,000	>100,000

^a^ Each cell line was infected with serial dilutions of various AAV serotypes expressing an EGFP reporter gene. Infectious titers were determined by evaluating EGFP-expressing cells after 3 days, apart from Anc80L65, where the EGFP-signal often increased until day 5. Thus, MOIs shown for Anc80L65 represent day 5. The MOIs depicted here display the lowest MOI with a significant fluorescence, showing an infectivity of ≥50%. AAV serotypes that are highly efficient at infecting a particular cell line are shown in bold.

**Table 3:** Percentage cleavage efficiency of FeLV provirus at target sites after transfection.

Target	CRFK			FEA			PG-4		
	TIDE	FA ^1^	Gel	TIDE	FA	Gel	TIDE	FA	Gel
T1	8.2	8.1	0.0	10.5	10.7	7.5	4.6	4.5	5.9
T2	21.3	14.6	15.1	27.5	16.1	8.3	12.2	7.6	14.0
T3	11.8	7.3	12.8	9.8	6.3	5.1	9.1	3.6	1.5
T4	62.6	16.9	28.6	60.8	24.6	30.0	41.3	21.0	22.5
T5	57.2	29.9	30.0	81.5	43.7	25.2	31.5	25.6	14.0
T6	42.5	21.6	27.2	45.6	17.6	16.3	16.9	20.0	18.8
T7	68.0	13.3	14.0	74.5	12.0	10.6	39.0	26.2	15.1
T8	79.4	10.0	26.5	85.8	30.0	23.8	38.4	28.5	14.6
T9	10.1	6.7	18.8	26.0	11.7	11.7	9.8	10.8	11.7
T10	43.7	14.4	14.6	48.6	12.3	9.4	34.3	12.1	9.4
T11	N.A.	N.A.	N.A.	40.1	17.1	12.3	14.1	7.1	6.2
T12	21.4	13.7	9.4	17.7	8.8	8.9	8.9	6.7	10.0

^1^ FA: Fragment Analyzer; N.A. not available.

**Table 4:** In vitro cleavage of FeLV provirus at target sites after transduction.

Target		CRFK			FEA			PG-4	
	TIDE	FA ^1^	Gel	TIDE	FA	Gel	TIDE	FA	Gel
T4 (vT4)	49.17	53.63	39.18	12.17	27.71	28.86	13.30	23.83	27.37
T5 (vT5)	58.90	43.25	31.86	34.27	41.14	36.30	18.30	29.03	34.94
T6 (vT6)	32.07	35.48	25.23	13.00	16.07	15.58	4.43	7.62	15.98
T8 (vT8)	**81.60**	37.15	40.97	47.87	43.54	35.92	44.50	44.96	34.25
T10 (vT10)	32.00	17.48	18.41	7.50	5.26	13.33	2.73	**1.18**	5.96

The mean frequency of indels of the triplicates is depicted. ^1^ FA: Fragment Analyzer. In bold are the highest and lowest percentages of NHEJ.

## Data Availability

All available data are presented in this manuscript.

## Supplementary Materials

The following are available online at https://www.mdpi.com/article/10.3390/v13081636/s1, Figure S1: comparison of FeLV provirus loads after transduction in CRFK (**A**), FEA (**B**), and PG-4 (**C**) cells. FeLV provirus loads measured by qPCR were compared after CRISPR/*Sa*Cas9 transduction, using the AAV-DJ vector without target (v426), Target 4 (vT4), Target 5 (vT5), Target 6 (vT6), Target 8 (vT8), Target 10 (vT10), and non-transduced cells (negative). The FeLV provirus load was normalized to copies/cell using fALB. The data are shown as box plots, and the boxes extend from the 25th to 75th percentiles. The horizontal line represents the median, and the whiskers extend from the smallest to the largest value. FeLV provirus loads were tested by Kruskal–Wallis, one-way ANOVA by ranks (p*_KW_* as indicated), and subsequently by Dunn’s post-test: * = *p* < 0.05. Table S1: medium ingredients.
