# Supplementary material for: Adeno-Associated Vector-Delivered CRISPR/SaCas9 System Reduces Feline Leukemia Virus Production In Vitro
Source: Viruses. 2021 Aug 18;13(8):1636. doi: 10.3390/v13081636 (PMC8402633; doi:10.3390/v13081636)
Supplement: Supplementary file 1 [file viruses-13-01636-s001.zip › viruses-1290924-supplementary.pdf]

viruses

MDPI

Supplementary Materials: Adeno-Associated Vector-Delivered CRISPR/*Sa*Cas9 System Reduces Feline Leukemia Virus Production In Vitro

Table S1. Medium ingredients.

FEA	advanced RPMI 1640[a]; 10% FCS[b]; 1% A/A[c]; 2 mM L-glutamine[d]
CRFK	advanced RPMI 1640[a]; 10% FCS[b]; 1% A/A[c]; 2 mM L-glutamine[d]
PG-4	McCoy's 5a[e]; 10% FCS[b]; 1% A/A[c]; 2 mM L-glutamine[d]; 1% NEAA[f]
Fc3Tg	EMEM[g]; 10% FCS[b]; 1% A/A[c];2 mM L-glutamine[d]: 1% NEAA[f]
Fcwf-4	advanced RPMI 1640[a]; 10% FCS[b]; 1% A/A[c];2 mM L-glutamine[d]
CAT-MT	EMEM[g]; 10% FCS[b]; 1% A/A[c];2 mM L-glutamine[d]
AKD	Ham's F12 [h]; 10% FCS[b]; 1% A/A[c];2 mM L-glutamine[d]; 1% NEAA[f]
FetJ	advanced RPMI 1640[a]; 10% FCS[b]; 1% A/A[c]; 2 mM L-glutamine[d]
PBMC	advanced RPMI 1640[a]; 10% FCS[b]; 1% A/A[c]; 2 mM L-glutamine[d]
HEK-293T	advanced RPMI 1640[a]; 10% FCS[b]; 1% A/A[c]; 2 mM L-glutamine[d]
Mus dunni	advanced RPMI 1640[a]; 10% FCS[b]; 1% A/A[c]; 2 mM L-glutamine[d]

[a] advanced RPMI 1640 (12633012, Thermo Fisher Scientific, Fremont, USA); [b] fetal calf serum (FCS; 16000044, Thermo Fisher Scientific); Gibco® Antibiotic-Antimycotic (A/A) containing 1% (V/V) penicillin/ streptomycin (10378016, Thermo Fisher Scientific); [d] L-glutamine (25030081, Thermo Fisher Scientific); [e] McCoy's 5A (Thermo Fisher Scientific); [f] non-essential Amino Acid (MEM NEAA; PAN Biotech, Aidenbach, Germany); [g] EMEM (EBSS; Thermo Fisher Scientific).

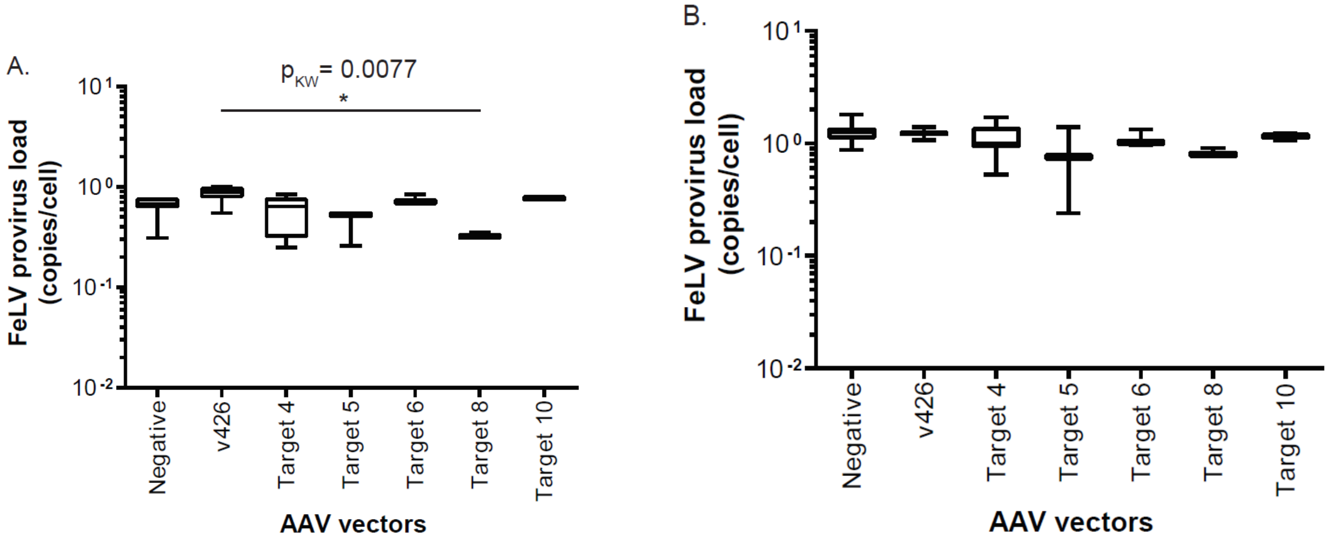

(**a**) (**b**)

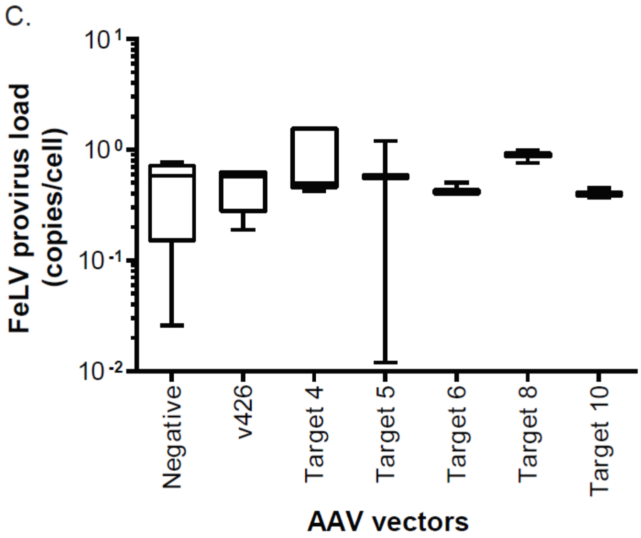

(**c**)

Figure S1. comparison of FeLV provirus loads after transduction in CRFK (**A**), FEA (**B**), and PG-4 (**C**) cells. FeLV provirus loads measured by qPCR were compared after CRISPR/*Sa*Cas9 transduction, using the AAV-DJ vector without target (v426), Target 4 (vT4), Target 5 (vT5), Target 6 (vT6), Target 8 (vT8), Target 10 (vT10), and non-transduced cells (negative). The FeLV provirus load was normalized to copies/cell using fALB. The data are shown as box plots, and the boxes extend from the 25th to 75th percentiles. The horizontal line represents the median, and the whiskers extend from the smallest to the largest value. FeLV provirus loads were tested by Kruskal–Wallis, one-way ANOVA by ranks (p_{KW} as indicated), and subsequently by Dunn's post-test: * = $p < 0.05$. .
